# Adapting to Climate Change: Leveraging Systems-Focused Multidisciplinary Research to Promote Resilience

**DOI:** 10.3390/ijerph192214674

**Published:** 2022-11-08

**Authors:** Sara M. Amolegbe, Adeline R. Lopez, Maria L. Velasco, Danielle J. Carlin, Michelle L. Heacock, Heather F. Henry, Brittany A. Trottier, William A. Suk

**Affiliations:** 1 Superfund Research Program, National Institute of Environmental Health Sciences (NIEHS), National Institutes of Health (NIH), Department of Health and Human Services (HHS), Durham, NC 27709, USA; 2 MDB, Inc., Durham, NC 27713, USA

**Keywords:** climate change, multidisciplinary, systems approach, resilience, Superfund

## Abstract

Approximately 2000 official and potential Superfund sites are located within 25 miles of the East or Gulf coasts, many of which will be at risk of flooding as sea levels rise. More than 60 million people across the United States live within 3 miles of a Superfund site. Disentangling multifaceted environmental health problems compounded by climate change requires a multidisciplinary systems approach to inform better strategies to prevent or reduce exposures and protect human health. The purpose of this minireview is to present the National Institute of Environmental Health Sciences Superfund Research Program (SRP) as a useful model of how this systems approach can help overcome the challenges of climate change while providing flexibility to pivot to additional needs as they arise. It also highlights broad-ranging SRP-funded research and tools that can be used to promote health and resilience to climate change in diverse contexts.

## 1. Introduction

Climate change—perhaps the single biggest health threat facing humanity, according to the World Health Organization and the United Nations [1]—affects human health and wellbeing in many ways. Changing environmental conditions, including rising temperatures, droughts, floods, wildfires, and other extreme weather events, increase exposure to heat, introduce new pests and pathogens, and strain infrastructure systems such as healthcare [2,3,4].

Climate change conditions also alter the movement, bioavailability, and toxicity of hazardous substances in the environment, and thus people’s exposure to these substances [5]. For example, higher temperatures may alter the biotransformation of contaminants into more biologically active metabolites, and areas experiencing less rainfall may contribute to higher volatilization of contaminants, while areas receiving greater precipitation may experience more contaminant runoff or deposition of airborne contaminants [6].

Of particular concern are the roughly 10,000 contaminated sites regulated under the U.S. Environmental Protection Agency Superfund Enterprise Management System (SEMS), which includes more than 1500 Superfund National Priorities List (NPL) sites [7,8]. These active Superfund sites, proposed sites, and those in the screening and assessment phases contain hundreds of harmful chemicals, including pesticides, chlorinated solvents, dioxins, and others [9]. 

Federal data suggest approximately 60% of nonfederal Superfund National Priorities List sites are in areas that may be impacted by wildfires and different types of flooding [10]. Similarly, approximately 2000 SEMS sites are located within 25 miles of the East or Gulf coasts, many of which will be at risk of flooding as sea levels rise [7]. Under high rates of sea level rise, more than 1000 Superfund sites could be at risk of extreme coastal flooding by the year 2100. Even at low rates of sea level rise, as many as 800 of these sites would still be at risk of flooding in the next 20 years, with many others susceptible to additional climatic effects. 

More than 20 million people across the United States live within a mile of a Superfund site, and more than three times that many live within 3 miles [11]. Superfund sites often exist near lower-income neighborhoods and communities of color [7,11,12]. Specifically, 28% of all minorities in the United States and approximately 25% of all households below the poverty line live within 3 miles of a Superfund site [8]. 

Environmental justice literature shows that racial or ethnic minorities and people of low socioeconomic status bear a higher burden of exposure to toxic waste and other environmental hazards, as well as social stressors, such as poor housing, access to health services, poverty, and racial discrimination [13,14,15]. These environmental and social stressors have synergistic and cumulative effects on the health of the populations, resulting in environmental health disparities [13]. Studies also show that climate change will reinforce and amplify current and future health disparities [16,17]. 

In addition to changing direct exposure risks from hazardous substances at Superfund sites, climate change also adds additional stressors that can affect health, such as exposure to extreme heat, increased exposure to infectious agents, threatened food and water supplies, increased burden of disease, or emotional and psychological distress [18,19,20]. 

A growing body of research highlights how combined exposures to mixtures of contaminants and social determinants of health—social inequities in the places where people live, work, or play that can lead to traumatic experiences, physiological stress, sickness, and health disparities—interact to adversely affect health [21,22,23,24,25,26,27]. As extreme weather events become more frequent and intense, experts anticipate existing health disparities will worsen and new public health challenges will arise as these factors converge [7,18]. 

Because climate change affects many aspects of the interconnected natural, built, and social environments, all are increasingly vulnerable to cascading effects that are often difficult to predict [28]. A solution-oriented systems approach—which offers a framework for teams of scientists to integrate diverse research toward addressing a specific environmental health question as part of a larger perspective—is critical to advancing research on the intersection of climate change and human health [29]. Disentangling multifaceted environmental health problems compounded by climate change can provide new insights that inform better remediation approaches, new targeted therapies, and sustainable strategies to prevent or reduce exposures that can harm human health. 

The purpose of this minireview is to present the National Institute of Environmental Health Sciences Superfund Research Program (SRP) as a useful model of how a multidisciplinary, systems-focused approach can help overcome these challenges, with the flexibility to pivot to additional needs as they arise. It will also highlight the broad range of research and tools that others can leverage to help address climate change in diverse contexts. 

## 2. Importance of Multidisciplinary Research

Addressing the multifaceted problems posed by climate change requires engaging with communities who are directly and disproportionately affected. This requires multidisciplinary and multisectoral partnerships, collaborative data integration, and rigorous training to equip the next generation of environmental health leaders with diverse and complementary skills [30,31,32,33,34,35,36], all hallmarks of SRP. 

As part of the National Institutes of Health, SRP was established in 1986 under the Superfund Amendments and Reauthorization Act. Driven by broad, cross-cutting mandates [37], SRP funds interdisciplinary, university-based research to evaluate the health effects of exposures to contaminants at hazardous waste sites and to provide solutions to protect human health. 

**Figure 1:**
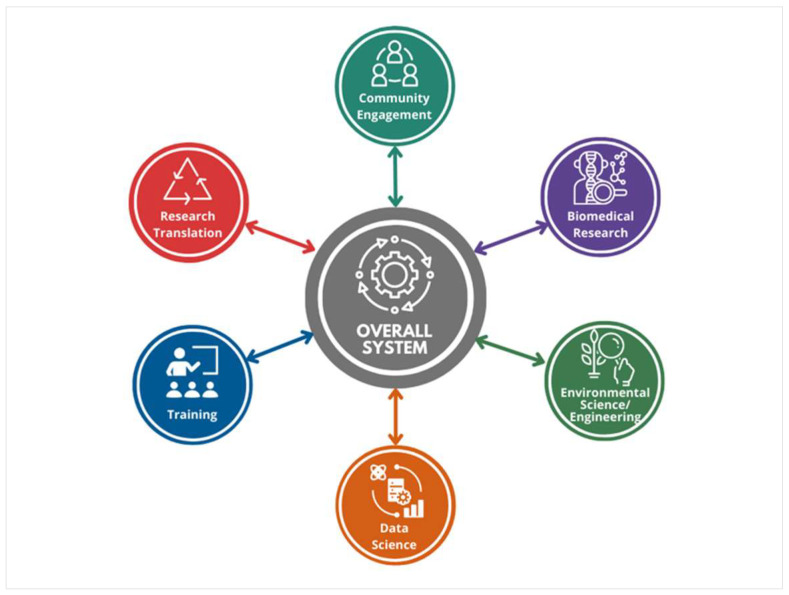
SRP-funded multiproject centers combine biomedical research, environmental science and engineering, research translation, community engagement, training, and data science to understand and address a specific problem. Leveraging a systems approach can advance SRP science that converges across disciplines, while building the foundation for researchers to address difficult emerging environmental health problems, such as climate change.

SRP connects researchers normally siloed in distinct disciplines to create bridges between basic biomedical research, public health, and environmental science and engineering. Researchers integrate expertise across these broad disciplines and work with state and local agencies, tribal governments, nonprofit organizations, and communities impacted by hazardous waste to address specific environmental health questions as part of a larger system (Figure 1) [29]. 

Together, these multidisciplinary teams are developing advanced sampling techniques, using innovative modeling approaches, designing sustainable cleanup strategies, and working closely with affected communities to protect against environmental hazards. This research model has uncovered new ways of thinking about complex problems, resulting in substantial scientific, public health, and economic benefits [38,39]. 

Because these projects generate a wealth of data, SRP has embraced a culture of data sharing to enable multidisciplinary, systems-focused centers to better identify innovative solutions to difficult environmental health problems while accelerating the pace of research in new areas [29,40,41]. For example, as teams across SRP centers collaborate to combine and integrate their diverse data, they are able to reveal new scientific connections and a more comprehensive understanding of the interplay between exposures and health, from better understanding population-specific sources of exposure to contaminants [42] to predicting how people may be exposed to contaminants during flood events [43,44].

By leveraging robust research and training infrastructure, as well as partner networks, and collaborating to combine data from diverse disciplines across research projects and centers, SRP grantees are well positioned to predict and address emerging and evolving challenges associated with climate change while developing new tools and strategies that can be adapted by others to promote sustainability, health, and resilience.

## 3. SRP Climate Research: Past, Present, and Future

**Figure 2:**
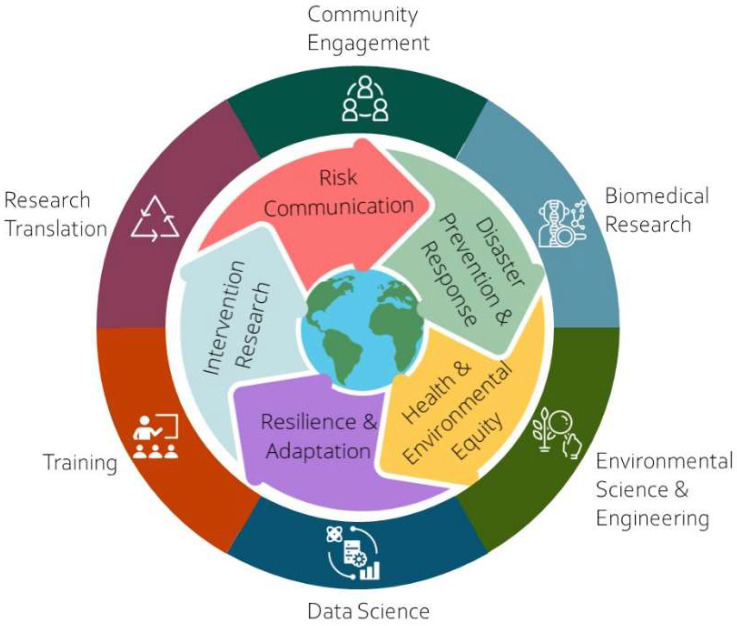
SRP grantees work across multidisciplinary teams to develop advanced sampling techniques, use innovative modeling approaches, design sustainable cleanup strategies, and work closely with communities to promote health and resilience in the context of climate change.

As part of its research portfolio, SRP supports research to better understand and address the effects of flooding, fire, land use changes, and other disaster events on exposure to hazardous substances and effects on human health (Figure 2). As a rapidly evolving area, and an essential element of federal policies and national security planning [45], the volume of research in this area will continue to grow.

**Figure 3:**
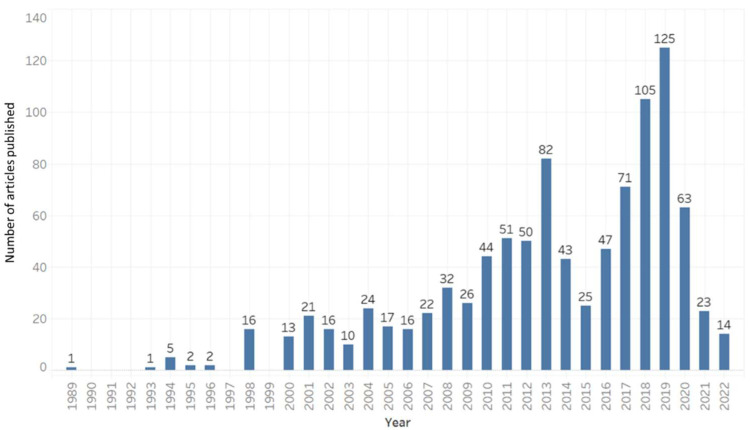
Bar chart depicting the growth in SRP grantee publications related to climate change over time. Note: historical publications are more difficult to attribute to SRP funding as grant reporting requirements for publications were not in effect, thus earlier data are likely an underestimate SRP contributions.

**Figure 4:**
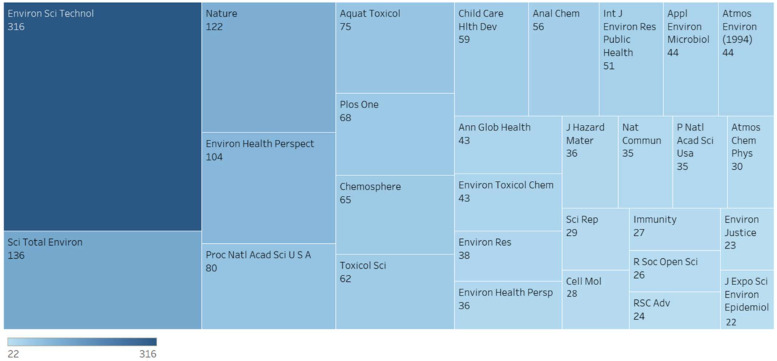
Top journals where SRP grantees have published climate change-related work and number of articles. This image illustrates the breadth and diversity of SRP grantee research within this area, including toxicology, epidemiology, environmental justice, environmental chemistry, and more.

**Figure 5:**
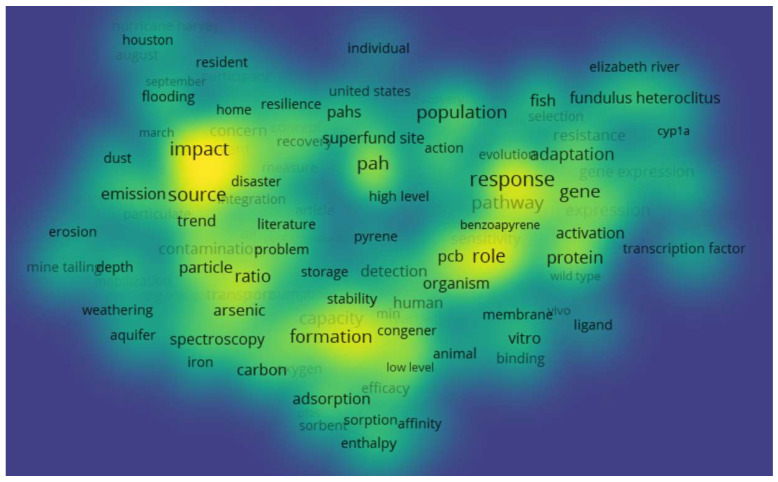
Density map of title and abstract keywords for all SRP grantee publications related to climate change (all time) generated in VOSviewer. A larger font size and yellow color indicate a higher frequency of keyword occurrence. The distances between each of the keywords indicate the relatedness of the research topics.

SRP grantee research in this area is broad and cuts across many scientific disciplines (Figure 3). Together, these activities attempt to tackle the same overarching goal, to protect human and environmental health. Over the years, grantee research in this space contributed to new understanding of key issues and common themes to explore further (Figure 4 and Figure 5). 

The examples below highlight SRP grantees’ solution-oriented research that can be used to inform health-protective decisions to promote resilience—the ability to prepare for, recover from, and adapt to the impacts of climate change. From understanding and predicting changing exposures, to developing sustainable solutions to remove contaminants from the environment, understanding potential effects on human health, and engaging with affected communities—such as by involving them in research and sharing tools and knowledge—SRP grantee research enables people to reduce their exposures and protect health in the face of climate change. 

## 4. Understanding Contaminant Movement in a Changing Climate

Chemical pollutants move and change in the environment as a function of their physical and chemical characteristics and local conditions, including the presence of other co-occurring chemicals or nutrients, soil and water properties, wind and precipitation, and microbial communities. To protect health, scientists and policy makers need information on how climate change affects contaminant transport and human exposure, in order to develop approaches to adequately assess and manage health risks [5]. 

Significant changes in weather characteristics, including precipitation, temperature, and wind patterns, will affect virtually every corner of the globe [2]. While flooding is expected to become more common in some regions and hurricanes are likely to become more intense, many areas that are currently semiarid are projected to experience more prolonged periods of drought and more intense wildfires [28]. Both scenarios have important implications for how chemicals are distributed and transported in the environment and how people are exposed. 

SRP researchers have developed and used cutting edge tools to detect contaminants in air, water, and soil, during and after a variety of disasters including Hurricane Florence in North and South Carolina [46,47,48], Hurricane Maria in Puerto Rico [49,50], and wildfires [51,52]. They have also documented direct and indirect health effects following hurricanes [53,54]. The case study below focuses on Hurricane Harvey and provides an example of one of the many research activities in this area. 

### Redistributed Contaminants from Hurricane Harvey

When Hurricane Harvey made landfall in Houston in 2017, Texas A&M University (TAMU) SRP Center researchers quickly mobilized [55]. They took advantage of their recent collaboration with a community partner organization, Texas Environmental Justice Advocacy Services (T.e.j.a.s.), and the trusting relationships they had already built within the community. T.e.j.a.s. organized public meetings with community members, state officials, and local nonprofits, in order to understand community needs and build resilience. The researchers also trained community scientists to collect data and participated in monthly community meetings. 

Together, the team collected environmental samples from neighborhoods surrounded by refineries, freeways, a rail yard, and water treatment facilities, and compared them to samples collected just before the storm to characterize how flooding may increase exposure to certain contaminants [56]. They also collected samples near the San Jacinto Estuary and found that Hurricane Harvey eroded sediment contaminated with mercury, resulting in at least two tons of mercury being released into Galveston Bay [57].

In collaboration with an SRP-funded researcher from the Virginia Institute of Marine Sciences, TAMU scientists field-tested a sophisticated biosensor [58], demonstrating its utility for quickly characterizing and prioritizing environmental samples for further analysis [59]. This tool may be particularly useful in the context of disaster research response where time and resources are limited. 

They also demonstrated how in vitro bioactivity data from post-disaster sediment samples can be used as a comprehensive indicator of potential hazards to inform risk management and remediation of affected areas [60].

In related work, the TAMU and Baylor College of Medicine SRP centers collaborated to collect personal exposure data after the hurricane. The team deployed silicone wristbands created by scientists at the Oregon State University SRP Center to measure the chemicals people were exposed to in the Houston area [61,62]. The researchers analyzed 173 wristbands, worn by people living or working in flooded areas for a week, and found 183 chemicals, most of which are known to interfere with the endocrine system [63]. They reported that chemical levels were generally higher after the hurricane compared data collected one year later [64]. The simple, non-invasive wristbands effectively measure personal exposures to hundreds of different chemicals, and have been successfully deployed in diverse settings [65,66,67,68]. 

Finally, the team collaborated with researchers at the UC San Diego and Brown University SRP Centers to develop an online interactive dashboard to display how land use, such as green space or industrial land, interacts with extreme weather to affect public health [43,69]. They demonstrated how integrating data can help identify neighborhoods threatened by contaminant redistribution during flooding events [43]. While this tool was originally developed for San Diego County in California, Harris County in Texas, and the state of Rhode Island, the approach can be modified and used for other visualizations of elements affecting human health in different locations to help community members make informed decisions to reduce or prevent environmental risks.

## 5. Sustainable Approaches to Cleaning up Pollutants in the Environment

Removing hazardous substances from the environment is critical to preventing exposure and reducing effects on the environment and human health [70]. The complex interplay between climate change and contaminant fate and transport can affect how remediation approaches used to clean up contaminants are applied and how effective they are. These factors and the challenges faced by the affected communities must be considered during planning and development to proactively promote strategies that are resilient to uncertain environmental conditions [71]. 

Unexpected, climate-driven conditions (e.g., droughts, storm events, permafrost melt, wildfires) can introduce new contaminants to sites, release formerly stable contaminants into groundwater or soil, damage containment infrastructure, and introduce changes to vegetation, adding another layer of complexity to ongoing cleanup efforts. Rising temperatures can also make certain contaminants more volatile [72]. Some remediation strategies are more sustainable and resilient to extreme weather events than others. For example, conventional soil capping, which uses materials like sand, silt, and clay, may result in contaminant releases during floods or heavy rains [73]. 

In addition, traditional remediation approaches, such as pump-and-treat and electrokinetic remediation, often use other chemicals, and can be energy-, time-, and cost-intensive [74]. Sustainable remediation methods focus on developing technologies or methods that are faster and less expensive, have a lower carbon footprint, take better advantage of renewable resources and energy, and have better regeneration capacities compared with existing approaches [75]. These strategies will be beneficial as the climate continues to change. 

SRP researchers have developed innovative, sustainable strategies to clean up contaminants in the environment, including technologies like functionalized nanofibers to remove uranium from water [76], self-cleaning water purifiers for low-resource settings [77,78], and renewable PFAS filters that have been adapted into household water filter pitchers [79]. Scientists are also transforming and reusing contaminated urban storm water [80,81], and scaling up PCB degradation under realistic conditions [82]. The case studies below illustrate some of the many research advances in this area in more detail.

### 5.1. Promoting Vegetation Cover

SRP researchers from the University of Arizona explore how promoting vegetation cover at mining wastes sites in arid environments can stabilize contaminants and prevent their transport into the environment, such as by wind-blown dust [83], reducing or preventing the risk of exposure to humans. 

The team found that increasing temperatures, drought, and gap soils in these ecosystems may decrease microbial and vegetation density, compromising the capacity of the soil microbiome to sustain necessary functions for plant growth and development and negatively impacting remediation [84,85]. To address these challenges, researchers are studying how plants interact with microbes and metals in contaminated soils to improve their ability to take up metals from mine waste sites [86]. They are also collaborating with the University of California (UC), San Diego SRP Center to develop genetically engineered plants that can resist droughts and grow in semi-arid environments. 

### 5.2. Promoting Drought and Salinity Tolerance

Scientists from the UC San Diego SRP Center aim to improve plant resilience in the face of climate change by exploring plants’ biological responses to stress, such as stomatal closure to prevent water from escaping in drought conditions [87,88] and elevated atmospheric carbon dioxide levels [86,89]. 

They collaborated with researchers at the Dartmouth College SRP Center to identify key transporters in plants that can increase the uptake of water and nutrients in soil and improve the ability of crops to resist environmental stressors, including salinity, pathogens, and toxic aluminum levels [90]. The Dartmouth team studied the mechanisms by which plants adapt to salinity in coastal environments to improve plant resilience with a rise in sea levels [91] and the roles of iron metabolism and the root microbiome in how plants adapt to drought [92]. These projects can help inform future efforts to improve drought tolerance by shedding light on the relationship between plants, microbes, and nutrients in soil.

### 5.3. Innovative Technologies

SRP research projects, funded through an individual research grant mechanism [93], are incorporating advances in material science to optimize bioremediation of contaminants in soil, sediment, or water. These projects may offer exciting breakthroughs to advance sustainable solutions for removing hazardous substances from the environment. 

For example, investigators are developing nanomaterials that can remove contaminants such as arsenic and per- and polyfluoroalkyl substances from soil [94]. Others are using solar electricity to accelerate the activity of bacteria to clean up halogenated contaminants. Still others are using nanotechnology to speed up the growth and enhance the activity of fungi that break down persistent organic pollutants [94]. 

## 6. Understanding Effects and Protecting Health

Communities often struggle to prepare for, respond to, and recover from climate change-related events such as storms, floods, and heat waves, which can affect human health and community resilience [95]. Climate change can directly affect human health, such as through disaster-related injuries and heat-related illness, and through wide-ranging indirect effects. Indirect effects can include threatened food supplies, increased distribution of infectious diseases, strained mental and physical health, and worsened air and water quality [18,19,20]. 

SRP-funded researchers, in collaboration with community partners and other stakeholders, are revealing how climate disasters, and exposure to harmful chemicals redistributed during these events, affect people’s well-being. For example, altering the amount of contaminants that the body can absorb [96], triggering neurological problems and premature aging [97], and straining mental health [98]. Importantly, researchers are also identifying sustainable strategies to adapt and promote community resilience, including developing edible sorbents that reduce exposure to and toxicity from hazardous substances following natural disasters [99,100,101]; providing disaster response training [102,103]; and exploring land use, green infrastructure, and development to promote community and ecological resilience [104,105,106,107,108,109]. The case studies below provide more detailed examples of the many research activities in this area.

### 6.1. Meeting Basic Needs and Exploring the Effects of Hurricane Maria 

Since 2010, researchers from the Northeastern University SRP Center have sought to understand the link between exposure to hazardous chemicals and preterm birth among women in Puerto Rico. To accomplish this goal, the team has developed strong bidirectional engagement with community stakeholders, including study participants, broader community groups, and clinicians. Through public meetings and focus groups, they identify community priorities, needs, and concerns, while refining their approach for reporting back individualized study results to participants [110]. 

When Hurricane Maria devastated the island in 2017, the team quickly mobilized, leveraging their existing infrastructure and trusted partner networks to meet the basic health needs of their community [111,112,113]. Since then, they have continued to investigate the effects of Hurricane Maria on people’s health in Puerto Rico. 

For example, they found harmful chemicals in excess of regulatory levels in tap water [50] and higher levels of phthalates commonly found in food packaging in pregnant women [114] after the hurricane. According to the team, changes in chemical exposures and stress, widespread structural damage, food and water shortages, and lack of electricity and cell phone service may all play a role in increasing the risk of adverse birth outcomes among this already vulnerable population. 

The researchers also examined data on childbirth and weather in Puerto Rico between 1994 and 2012, reporting that extreme weather events and changes in temperature and precipitation increase the risk of preterm birth [115]. Combined with other evidence from this cohort that psychosocial stress can worsen the effects of exposure to hazardous substances [116,117], the team is adding to our understanding of the complex relationship between health and exposome—that is, the totality of exposures across the course of life, including chemicals, stress, diet, and more. 

They also evaluated the impacts of hurricanes Irma and Maria on conducting children’s environmental health research [118], revealing the need to integrate disaster preparedness into similar research programs to promote resilience among staff, community health workers, and participants.

### 6.2. Promoting Resilience after Hurricane Harvey

Researchers at the TAMU SRP Center are using urban planning and citizen science, in which community members are trained to collect and analyze data, to make Houston communities more resilient to frequent flooding, air pollution, and health concerns stemming from natural disasters. For example, they worked with communities in the greater Houston area to develop a master plan for installing green infrastructure, such as water-absorbing rain gardens, on vacant lands [119]. Using a similar approach, they developed a community-scaled master plan for Manchester, Texas, to increase flood resiliency and decrease exposure to contaminants [120]. They projected that the plan could capture 147,456 cubic feet of runoff and create other benefits, such as reducing energy use and increasing carbon dioxide sequestration. 

The team also developed a regional growth framework to balance the need to repurpose and develop vacant lots while retaining critical ecosystem services [121]. Their approach can identify vacant lands with low development potential and high ecological value, pinpointing ecological corridors that can be protected with minimal impact on development or the local economy. 

Using a case study with different scenarios of green infrastructure, the group reported significant reductions in storm water runoff and pollutants compared with the baseline [122]. Similarly, using historical flood data and changes in green infrastructure over time, they were able to identify an optimal strategy involving protecting larger green spaces near flood prone areas to reduce the costs associated with flood damage [123]. These tools can be adapted to better understand how to prevent the harmful effects of climate change and promote resilience in different cities and regions.

## 7. Engaging Communities and Sharing Tools

For scientific research to have tangible effects on public health, researchers must engage with and seek input from communities, especially those disproportionately affected by environmental contamination and the effects of climate change. Even more critical is understanding the needs of and collaborating with underserved communities, such as minorities, low-income people, and those who live in remote or rural areas [124,125,126]. 

Through collaboration and knowledge transfer [127], SRP grantees are enhancing the local capacity to address environmental health challenges and promoting climate resilience by developing culturally relevant communication materials [128]; amplifying community voices [129]; and building awareness of specific exposures and their potential health effects, called environmental health literacy [130,131,132,133]. Others are implementing strategies that integrate social science and environmental health research, as well as citizen science data with traditional data sources, more holistically [54,124,134]. 

The examples below illustrate some of the many research activities in this area in more detail.

### 7.1. Engaging and Communicating with Affected Communities

Including communities disproportionately affected by environmental contamination in scientific research can spur social and environmental action to address environmental health disparities and increase community resilience in the face of climate change. SRP-funded researchers engage with diverse communities across the United States. They have uncovered common challenges and designed innovative approaches to tailor health messages for specific communities to enable them to act [135,136]. For example, University of Arizona investigators train community health workers about environmental health, climate change, and environmental monitoring protocols to then educate community members about these subjects. According to the team, these peer-education programs improve community knowledge about water and energy conservation, as well as give community members tools to turn scientific learning into environmental action [137]. 

SRP researchers also train community members on collecting and interpreting environmental data to identify exposure sources and develop their own risk-reduction recommendations in response to environmental disasters that may be exacerbated by climate change. In response to community concerns after the Gold King Mine Spill, the University of Arizona SRP Center initiated a citizen science project with the Navajo Nation to collect environmental samples to characterize soil and water impacts throughout the impacted region [138]. 

Engaging with communities also promotes equity in risk communication by ensuring that all individuals—regardless of race, nationality, or income—have access to and benefit from information. With community engagement, researchers can design more tailored messages and better translate research into communication tools that speak to the context.

### 7.2. Making Research Interactive and Actionable

Multidisciplinary teams of SRP researchers, including environmental health researchers, social scientists, and computational experts, create simple user-friendly communication and mapping tools to make science accessible and interactive to inform individual, community, and regulatory actions to protect human health. Many of these tools can be modified and adapted for different regions or populations. For instance, Boston University SRP Center researchers created a mapping tool to evaluate the threats posed by global warming, like flooding, to facilities that store toxic chemicals in the state [139]. Another mapping tool that may be useful in the context of climate change is MapGAM, which can help visualize how chemical and non-chemical stressors near the New Bedford Harbor Superfund site may alter risk patterns related to health outcomes, risk-taking and other behaviors, mental health, and community resilience [140,141,142]. 

Tools developed by the TAMU SRP Center can provide policy makers and others tasked with mitigating risks with detailed information about factors that contribute to vulnerability to environmental disasters, including climate-change-related events, such as flooding and sea level rise. For example, HGBEnviroScreen can help communities in the Houston–Galveston–Brazoria area in Texas understand environmental risk factors, including natural disasters, and identify and prioritize regions of heightened vulnerability [143]. The Toxics Mobility Vulnerability Index (TMVI) [69], which includes environmental, geographic, public health, and social factors, can show how land use, such as impervious surfaces and green space, contributes to flood risk [43]. 

Similarly, a team at the UC Berkeley SRP Center, in collaboration with a community partner, created a tool to provide communities with information about groundwater challenges that could affect their access to safe drinking water, such as unregulated wells, landfills, and climate-related events like sustained droughts [144,145].

After Hurricane Florence made landfall in North Carolina, Duke University SRP Center researchers updated a mapping tool showing potential sources of contamination in North Carolina to include hurricane-related incidents and floodplain data to help individuals reduce or prevent potential exposure to harmful chemicals [146]. The team also collaborated with the North Carolina State University Extension to create a website with resources to manage the impact of floodwater before, during, and after extreme weather events to help home gardeners protect themselves from contaminants [147].

The Digital Exposure Report-Back Interface (DERBI) [148], developed by the Silent Spring Institute and the Northeastern University SRP Center, allows scientists to report personalized exposure results, such as in the context of contaminant releases following disasters and climate change. For example, at the UC Berkeley SRP Center, a team is using the DERBI application to create personalized exposure reports for women firefighters after a fire event [149]. Researchers at the University of Rhode Island SRP Center are also using the application to communicate results of PFAS testing to private well owners on Cape Cod, Massachusetts [150]. 

## 8. Future Research Needs

Climate change is affecting the health of populations across the world, intensifying the need for a health-centered response that improves health and well-being. Adapting to climate change requires preventing human exposure to harmful substances, reducing vulnerability to climate hazards, and minimizing health risks related to climate change [151]. NIEHS and SRP have been leaders in supporting research to understand the connection between climate change and human health [152,153,154,155]. These activities are also in line with the NIH Climate Change and Health Initiative and Strategic Framework, a coordinated effort by NIEHS and other NIH Institutes and Centers to support research, training, and capacity building activities that address the climate change and health crisis on a global scale [35] and Executive Orders [45]. SRP’s systems-focused multidisciplinary centers (see Figure 1) can serve as a model for how best to integrate diverse research surrounding a common, complex problem such as climate change to better understand the larger picture and identify solutions [29,40,41]. Much of the existing research, tools, and infrastructure developed by SRP grantees, such as new sampling and analytical techniques and advanced methods, can be adapted and scaled up to address climate change issues in different contexts to protect public health. Similarly, grantees’ insights into sustainable and climate-resistant strategies to address and clean up pollutants can inform better approaches to reduce the amount and toxicity of hazardous substances in a dynamically changing environment. 

SRP grantees are exploring how climate change worsens existing health threats and creates new physical and mental health risks, uncovering additional layers to the exposome that can be leveraged to identify solutions, such as informing health-protective practices and policies. By investigating the interplay between land use, weather, and social and behavioral factors, they are revealing new ways cities can better plan for climate resilience without stifling growth and economic development. 

Continued emphasis on rigorous, multidisciplinary training will help equip the next generation of scientific leaders with the skills and tools needed to work towards sustainable solutions and promote resilience in the face of climate change. In addition, expanding partnerships across sectors will ensure that innovative strategies are responsive to community needs and can be effectively put into practice to protect human health. 

Finally, SRP grantees engage directly with diverse communities affected by both environmental contamination and climate change. Their tools can be adapted for diverse populations and their experiences designing culturally relevant communication materials and building local capacity can help other groups more quickly identify and overcome barriers to effective risk communication and adaptive measures within their specific communities. 

## 9. Conclusions

As the climate continues to change, worsening existing health disparities and inequities among vulnerable populations, SRP grantees are well positioned to leverage existing research and infrastructure to continue identifying solutions to this and other emerging environmental health problems, while serving as a model for how multidisciplinary research centers can meet the challenges of a changing climate. 

By continuing to explore the complex interplay between climate change and health, scientists will reveal critical insights to inform robust health-protective climate policies and new strategies to promote resilience. Integrating expertise and data across disciplines, using a systems-approach framework, and involving community partners will help SRP grantees and others continue to develop effective tactics to reduce the health risks and advance research and education activities to protect public health.

## Data Availability

Not applicable.
